# Optimisation of Ultrasound-Assisted Extraction Conditions Using Response Surface Methodology and Identification of Thymoquinone from Black Cumin (*Nigella sativa* L.) Seed Extract^§^

**DOI:** 10.17113/ftb.63.02.25.8560

**Published:** 2025-06

**Authors:** Nita Kaushik, Aradhita Barmanray

**Affiliations:** Department of Food Technology, Guru Jambheshwar University of Science and Technology, Hisar-125001, Haryana, India

**Keywords:** ultrasonic extraction optimisation, thymoquinone identification, black cumin, FTIR analysis, GC-MS analysis, antioxidant potential

## Abstract

**Research background:**

*Nigella sativa* L., commonly known as black cumin, is a medicinal plant renowned for its rich bioactive composition and health-promoting properties. Among its key compounds, thymoquinone has gained significant attention in nutraceutical and pharmaceutical research for its potential to prevent and manage chronic inflammatory conditions and immune dysfunctions. With growing global interest in natural health solutions, the aim of this study is to optimise ultrasound-assisted extraction (UAE) conditions to maximise thymoquinone yield from the extract of black cumin (*Nigella sativa* L.) seeds and characterise the bioactive compounds. By using UAE and advanced analytical techniques, the research contributes to the development of sustainable extracts rich in bioactive compounds with applications in medicine and nutrition.

**Experimental approach:**

In this study, ultrasound-assisted extraction method was used with response surface methodology (RSM) software to extract the bioactive compounds, including total phenolic content (TPC) and compounds that can bind free DPPH radical. To increase the extraction efficiency of bioactive compounds, the following parameters were examined: the ratio of the mass of seed powder to the volume of solvent of 50–100 %, extraction temperature of 30 °C, amplitude of 30–60 % and extraction time of 30–60 min. Black cumin seed extracts were characterised using scanning electron microscopy (SEM), while gas chromatography-mass spectrometry (GC-MS) analysis was carried out to identify thymoquinone. Additionally, Fourier transform infrared (FTIR) spectroscopy confirmed the presence of thymoquinone and several functional groups, including amines, alkanes, acids, esters, alkyls and alkenes.

**Results and conclusions:**

Ultrasonic extraction using methanol as a solvent resulted in a higher yield of thymoquinone (28.62 %), identified using GC-MS analysis. The presence of thymoquinone was further confirmed by the functional groups detected in FTIR analysis. Under the specified extraction conditions, total phenolic content (TPC, expressed as gallic acid equivalents), yield (in %) and DPPH radical scavenging activity increased by approx. 271.03 mg/g, and 4.5 and 83.06 %, respectively. In addition to thymoquinone, thymohydroquinone was also identified based on its molecular mass, retention time and peak values. Thymoquinone, a natural and potent phytochemical, offers a range of therapeutic properties, including immune-enhancing potential.

**Novelty and scientific contribution:**

Thymoquinone is a bioactive compound found in black cumin seeds, known for its potent antioxidant and immunity boosting properties. This research was conducted achieve the best possible extraction conditions for bioactive substances. Additionally, the results support the potential of thymoquinone as a therapeutic agent to treat various health conditions. The novelty lies in the development and optimisation of extraction techniques to maximise the yield and bioactivity of thymoquinone, a compound renowned for its robust antioxidant and immune-modulating properties. This work uniquely bridges the gap between the traditional use of black cumin and modern scientific validation, and addresses global health priorities. The results emphasise the importance of *Nigella sativa* as a sustainable and natural source of health-promoting compounds, meeting the increasing demand for plant-based bioactive compounds in preventive healthcare. By characterising the extraction conditions and demonstrating therapeutic potential of thymoquinone, this study contributes to both the scientific literature and practical advances in the development of functional food and nutraceuticals.

## INTRODUCTION

Black cumin (*Nigella sativa* L.), an annual herb belonging to the Ranunculaceae family, is indigenous to the Mediterranean region, Northern Africa, Southern Europe and Southwest Asia (*1*, *2*). The western and northern regions of India are the most important areas for the cultivation of black cumin. These regions include Punjab, Himachal Pradesh, Madhya Pradesh, Bihar, West Bengal, Assam, Rajasthan, and Maharashtra (*3*). Black cumin is also known by various names worldwide, including kalonji, kalajeera, nutmeg flower, devil-in-the-bush, black caraway and Roman coriander. In ‘The Canon of Medicine’, Avicenna recognised it as a natural remedy within the ‘Tibb-e-Nabawi’ (similar to Unani and Tibetan systems of medicine), highlighting its use in treating a range of ailments (*4*). According to Dubey *et al.* (*5*), *Nigella sativa* is a bisexual herbaceous plant that grows to a height of 20–90 cm and produces single flowers with multiple seeds encased in inflated capsules. Black cumin seeds are rich in bioactive compounds that contain essential oils (35–50 %), volatile oils (0.13–0.39 %), alkaloids, steroids, phenolic compounds, terpenoids, and saponins (*6*). A study by Kaushik and Barmanray (*7*) found that *Nigella sativa* seeds contain moisture (7.06 %), crude protein (18.67 %), crude fat (45.09 %), crude fibre (7.33 %), ash (4.33 %) and total carbohydrates (17.51 %) on a dry mass basis. Black cumin seeds contain trace amounts of essential minerals, including calcium (2.63 μg/mL), potassium (1.94 μg/mL), zinc (1.24 μg/mL), copper (0.31 μg/mL) and magnesium (0.30 μg/mL) on a dry mass basis (*8*). The primary phytochemicals in black cumin seeds include thymoquinone, dithymoquinone, thymohydroquinone, carvacrol, *p*-cymene, sesquiterpene, thymol, 4-terpineol, longifolene, *t*-anethole and α-pinene (*9*). Thymoquinone, a bioactive compound from the seeds of black cumin, has gained significant attention due to its potent antioxidant, anti-inflammatory, anticancer and antimicrobial properties (*10*). Despite its pungent and bitter taste, black cumin has been used to flavour a variety of foods, including vegetables, bread, curries, pickles, condiments and savoury dishes (*11*, *12*). Black cumin seeds are not only an important ingredient in garam masala, but also one of the five spices in *panch phoran*, a traditional spice blend made from cumin seeds, black mustard seeds, fenugreek seeds and black cumin seeds (*13*, *14*).

According to Salehi *et al.* (*15*), cumin seeds have attracted great interest in the fields of food, cosmetics and pharmaceuticals. They are known for their therapeutic properties, including the treatment of digestive and respiratory disorders such as asthma (*16*), bronchitis, dysentery and stomach pain (*17*), as well as for their use as an insect repellent, for rheumatism and to improve kidney and liver function (*18*). Black cumin contains many bioactive substances (*19*), including thymoquinone, which has a number of beneficial effects, such as anticancer, antioxidant (*20*), antibacterial (*21*), antifungal, anti-inflammatory (*22*), antiviral (*23*) and immune-modulatory properties (*24*).

In this study, the aim was to maximise the amount of total phenolic compounds (TPC), antioxidant activity (DPPH method) and yield from black cumin seed extract by ultrasound-assisted extraction. The optimisation was conducted by RSM software using three independent variables (time, amplitude and the ratio of the mass of seed powder to the volume of solvent): 30–60 min, 30–60 % and 50–100 %, respectively. The best optimal conditions were determined to estimate the effective extraction of bioactive compounds and yield.

## MATERIALS AND METHODS

### Chemicals and reagents

Standard of thymoquinone and Folin-Ciocalteu reagent were purchased from Sigma-Aldrich Chemicals Private Limited, Merck (Bangalore, Karnataka, India), HPLC grade methanol and dimethyl sulfoxide (DMSO) were purchased from Merck (Darmstadt, Germany). Hydrochloric acid (35 %), sodium carbonate and aluminium chloride were purchased from HiMedia (Mumbai, Maharashtra, Inidia).

### Plant material and extract preparation

In November 2023, mature black cumin seeds of the *Nigella sativa* AN-1 variety were acquired from the Indian Council of Agricultural Research's National Research Centre on Seed Spices (ICAR-NRCSS) in Ajmer, Rajasthan, India. It was necessary to wash the black cumin seeds with deionised water to remove any debris or damaged seeds. After washing, the seeds were dehydrated on a drying tray with hot air at 35 °C. After drying, the seeds were ground into a fine powder and then stored at

-20 °C in a freezer (CHMK-50; Blue Star, Mumbai, India) for later use. The moisture content of the fresh seed powder, measured as *m*(seed powder)/*V*(solvent), was (2.2±0.4) %.

### Proximate analysis

The proximate values for moisture, crude fat, protein, dietary fibre and ash were analysed according to the standards approved by the AOAC International, as described by Khalid *et al.* (*25*). When the total amount of protein, fat, moisture, fibre and ash in black cumin seeds was subtracted from 100, the amount of carbohydrates present in the seeds was obtained. The validity of the results was checked by repeating each analytical method three times to ensure that it was accurate.

### Extraction procedure

The bioactive compounds were extracted from black cumin seeds using an ultrasound-assisted extraction method. First, a laboratory grinder was used to ensure that the ripe black cumin seeds were ground into a fine powder. The extraction was carried out by mixing *m*(powdered seed):*V*(methanol)=1:10 in a 500-mL beaker. Ultrasonic extraction was performed using an ultrasonic probe (Powersonic, Hwashin Tech. Company, Jakarta, Indonesia) (frequency 20 kHz, amplitude 30–60 %), ensuring that the probe was immersed in the seed-solvent suspension. The bioactive compounds were extracted at a constant temperature of 32 °C, and the process was timed for different durations between 30 to 60 min, depending on the experimental design. Ultrasonic energy was utilised to rupture the cell walls and facilitate the release of bioactive compounds from the seed matrix into the solvent.

Immediately after the extraction, the suspension was filtered through Whatman filter paper no. 1 to remove any solid residues that were present in the liquid extract. After that, the extract was concentrated by evaporating the solvent in a rotary evaporator (CR–200; Cyberlab Corporation, Frenchtown, NJ, USA) at 45 °C while the pressure was decreased. The resulting crude extract was freeze-dried at -50 °C in a lyophiliser (Alpha 2-4 LD Plus; Martin Christ, Osterode am Harz, Germany) for 12 h to remove any remaining moisture and preserve the bioactive compounds. Finally, the dried extract was stored in amber glass vials at 4 °C for further analysis. To ensure accuracy and reproducibility of the results, each procedure was carried out three time. Ultrasound-assisted extraction was used to evaluate thymoquinone content, TPC, DPPH radical scavenging activity and yield from black cumin seeds. The compounds were extracted at a constant temperature of 32 °C and a frequency of 20 kHz using a 2.0-cm diameter ultrasonic probe that was immersed in the suspension of the seeds and solvent (*26*). The sample to solvent ratio was maintained at 25 g of black cumin seeds per 250 mL of solvent, placed in a 500-mL beaker. After the ultrasonic treatment, Whatman filter paper no. 1 was used to filter the suspension to separate the liquid extract from the solid residues remaining in the solution. The solvent was then removed from the sample mixture using a rotary evaporator set to 45 °C to concentrate the extract. To preserve the bioactive compounds, the raw samples were freeze-dried at -80 °C for 12 h (*27*). After drying, the samples were carefully stored in amber glass vials at 4 °C to protect them from light and moisture, ensuring their stability for more thorough investigation.

### Determination of DPPH radical scavenging activity

To evaluate the DPPH radical scavenging activity, the method described by Mohammed *et al.* (*28*) was used. The DPPH was dissolved in methanol, which resulted in the preparation of a stock solution of 0.1 mM. To determine the antioxidant capacity of the black cumin seed extracts, 3 mL of the DPPH solution were mixed with 1 mL of the methanolic extracts at different concentrations. The resulting mixture was thoroughly shaken to ensure uniform distribution and then stored at room temperature (32 °C) in the dark, to prevent light interference, for 30 min. After incubation, the absorbance of the sample mixtures, together with a control sample, was measured at 517 nm using a UV-VIS spectrophotometer (G10S UV-VIS; Thermo Scientific, Thermo Fisher Scientific, Waltham, MA, USA). A comparison was made between the decrease in the absorbance of the test samples and the control (*29*), which enabled the calculation of the DPPH radical scavenging activity. The scavenging capacity of the black cumin seed extracts (in %) was calculated using the following equation:


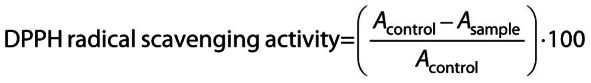
 /1/

where *A*_control_ is the absorbance of a methanolic solution containing DPPH and *A*_sample_ is the absorbance of a solution containing DPPH combined with extracts from black cumin seeds.

### Determination of total phenolic content

The Folin-Ciocalteu method was used according to Mohammed *et al.* (*28*) to determine the total phenolic content (TPC) of the black cumin seed extracts. A new stock solution of standard gallic acid was prepared to obtain a calibration curve. For each analysis, 1 mL of the appropriately diluted extract was combined with 0.5 mL of the tenfold diluted Folin-Ciocalteu reagent. After 5 min of incubation, 2 mL of a 20 % sodium carbonate solution were added to each tube. The final volume was adjusted to 10 mL with distilled water. To enable complete reaction development, the samples were then incubated at room temperature for 1 h in a light-restricted setting and a UV-VIS spectrophotometer (G10S UV-VIS; Thermo Scientific, Thermo Fisher Scientific) was used to measure the sample absorbance at 760 nm. To obtain a calibration curve, different concentrations of gallic acid standard solution were used. The total phenolic content was expressed in mL of gallic acid equivalents (GAE) per 100 g of dry sample mass. To ensure precision and reproducibility, all samples were analysed in triplicate and the results were reported as the mean value±standard deviation.

### Scanning electron microscopy image analysis

Scanning electron microscopy (SEM) was used to examine the morphological changes in black cumin seed matrices after ultrasound-assisted extraction. A mass of 1.0 mg of sample was meticulously affixed to aluminium stubs and sputter-coated with a tiny gold layer to improve conductivity. High-resolution images were taken at an appropriate accelerating voltage to capture detailed structural characteristics. SEM analysis provided high-definition images, enabling the visualisation of cell wall integrity, surface morphology and structural disruptions induced by ultrasonic treatment. This analysis was instrumental in evaluating the degree of cellular disintegration, thereby validating the efficiency of the extraction process.

### Gas chromatography-mass spectrometry analysis

The Shimadzu QP 2010 Ultra GC-MS chromatograph (Kyoto, Japan) with a Rxi®-5 Sil MS capillary column (20 m, 18 μm film thickness) was used to analyse the methanolic extract. After the sample had been diluted, it was injected using the standard mechanism and the flow rate of helium gas was maintained at 1 mL per min throughout the process. The oven was preheated for 5 min to a temperature of 70 °C. An electron energy of 70 eV and an ion source temperature of 200 °C were used. According to the results of the GC-MS analysis of the seed extracts, the presence of thymoquinone was confirmed.

### Fourier transform infrared spectroscopy characterisation

The spectra of thymoquinone were recorded using Fourier transform infrared spectroscopy (FTIR; Spectrum BX-II; PerkinElmer, Waltham, MA, USA). The spectra of thymoquinone showed distinct peaks of different intensities, indicating the presence of important functional groups such as C=O, C-H, -CH2, -CH3, C=C and C-O. This suggests that thymoquinone was well incorporated into the black cumin seed extract. A strong band at 2951.86 cm^−1^ indicates the C-H stretching of tertiary carbon group.

### Experimental design and statistical analysis

Statistical analysis was conducted using Design-Expert v. 13.0 software (*30*), a widely used tool for statistical design and analysis. A central composite design (CCD), which is a sort of response surface methodology (RSM), was used in this research to optimise the extraction of black cumin seed extract. The primary variables under investigation were extraction time (ranging from 30 to 60 min), ultrasonic amplitude (30–60 %) and the ratio of the mass of seed powder to the volume of the solvent (50–100 %). The purpose of the optimisation was to evaluate their effects on three key responses: total phenolic content (response 1), antioxidant activity using the DPPH method (response 2) and yield percentage (response 3). To ensure reliable and accurate results, each experiment was repeated three times, and twenty different iterations of the experiment were carried out in total, systematically varying the factors within the defined ranges. This approach enabled the determination of the most effective conditions for maximising the desired outcomes. Data collected from each experiment were reported as the mean value±standard deviation. To optimise the experimental settings and analyse the effect of different factors on the responses, statistical analysis was carried out using the Design-Expert v. 13.0 (*30*). A one-way analysis of variance (ANOVA) served to determine whether the differences between the experimental groups were meaningful from a statistical point of view. The p-value less than 0.05 (p<0.05) was considered statistically significant.

## RESULTS AND DISCUSSION

### Model fitting and RSM analysis

The effectiveness of ultrasonic extraction is significantly affected by different experimental parameters, including extraction time, ultrasonic amplitude and the ratio of the mass of seed powder to the volume of methanol. Table 1 summarises the responses of 20 experimental runs, designed using central composite design (CCD). It is well-established that parameters such as the duration of ultrasonication, the intensity of the ultrasonic waves, and the ratio of the mass of seed powder to the volume of solvent (methanol, in this case) play a crucial role in determining the efficiency of the extraction of bioactive compounds, including antioxidant activity, total phenolic content (TPC) and overall yield from seed extracts. For black cumin seed extracts, the results showed that the DPPH radical scavenging activity, TPC as GAE, and yield reached values of 83.06 %, 339.85 mg/g and 4.5 %, respectively. These findings emphasise the critical influence of extraction conditions on the recovery of bioactive compounds. Specifically, the optimisation of ultrasound-assisted extraction (UAE) parameters was shown to significantly improve the recovery of phenolic antioxidants. This highlights the importance of fine-tuning factors like ultrasonication time, amplitude and the mass of seed powder to the volume of the solvent to maximise the yield and antioxidant potential of black cumin seed extracts. These results also suggest that careful adjustment of ultrasonic extraction parameters can greatly improve the efficiency of the extraction of valuable compounds such as thymoquinone and other phenolic antioxidants from black cumin seeds. The study thus highlights the need to optimise the UAE process to achieve the best possible results in terms of both yield and antioxidant activity.

**Table 1:** Central composite design with coded variables and measured values

	Factor			Response		
Run	A: *t*/min	B: (*m*(seed powder)/*V*(solvent))/%	C: Amplitude/%	DPPH scavenging activity/%	TPC as *w*(GAE)/(mg/g)	*Y*/%
1	45	32.9552	45	33.67	235.65	4.3
2	45	75	70.2269	21.83	238.98	3.2
3	45	75	45	83.06	271.03	4.5
4	45	75	45	83.06	271.03	4.5
5	30	100	30	51.73	245.07	2.5
6	60	100	30	61.73	333.91	1.3
7	30	50	60	7.14	192.02	4.5
8	60	50	30	13.57	218.26	4.2
9	70.2269	75	45	28.67	300.2	2.8
10	30	50	30	3.87	339.85	3.7
11	45	75	45	83.06	271.03	4.5
12	45	75	45	83.06	271.03	4.5
13	19.7731	75	45	37.95	308.98	3.0
14	45	117.045	45	31.12	356.52	2.3
15	60	100	60	65.00	299.13	1.0
16	45	75	19.7731	41.02	301.44	3.3
17	45	75	45	83.06	271.03	4.5
18	45	75	45	83.06	271.03	4.5
19	30	100	60	46.22	312.46	1.3
20	60	50	60	18.57	278.26	3.0

DPPH=2,2-diphenyl-1-picrylhydrazyl radical, TPC=total phenolic content, GAE=gallic acid equivalent, =yield

### Scanning electron microscopy

**Fig. 1:**
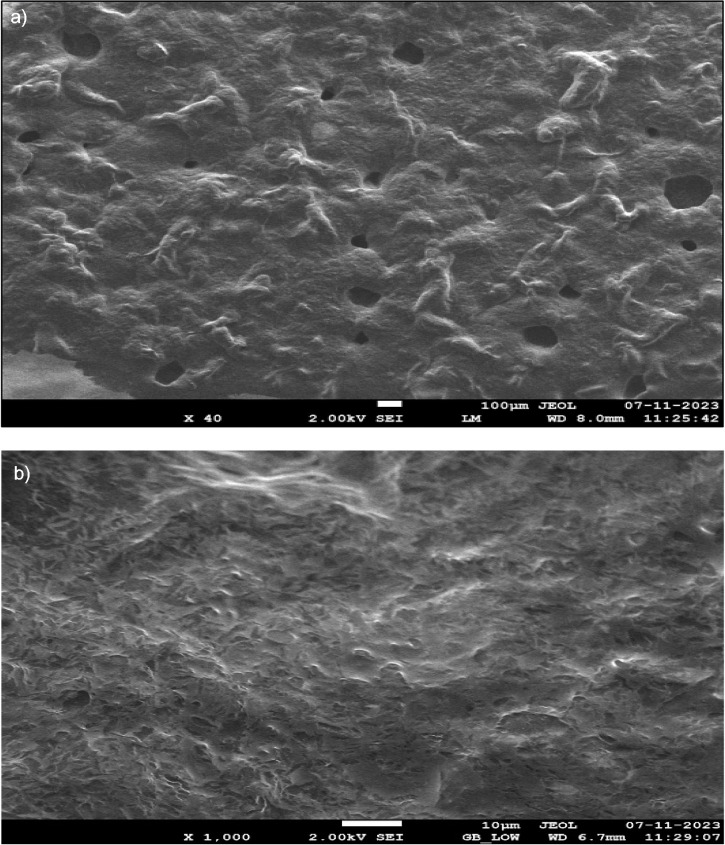
Image analysis of *Nigella sativa* extracts by scanning electron microscopy (SEM) using ultrasonic extraction at magnifications: a) 40× and b) 1000×

Fig. 1 shows the morphological changes observed in black cumin seed extracts after the ultrasound-assisted extraction. At 40× magnification (Fig. 1a), the surface of the seed matrix appeared relatively smooth, with minimal disruption. This suggests that the ultrasonic extraction did not cause significant surface damage. However, when the sample was further magnified to 1000× (Fig. 1b), the structural changes became more evident. The ultrasonic treatment caused noticeable surface cracking and fragmentation, resulting in an irregular, porous structure. The detailed cellular images clearly show that the ultrasonic waves caused significant disruption to the cell walls, and resulted in the formation of cracks and voids in the matrix. This structural disintegration indicates that sonication effectively breaks down the cell walls and increases the surface area, thus increasing the release of bioactive compounds. The images at higher magnification provide compelling evidence that the ultrasonic treatment leads to maximum cell disruption and loss of structural integrity. These results indicate that the ultrasound-assisted extraction is very efficient in promoting the release of valuable compounds from black cumin seeds. The increased porosity and cellular damage are likely factors contributing to the higher recovery of bioactive compounds such as thymoquinone, phenolic compounds, and other antioxidants. Therefore, it can be concluded that sonicated samples provide the maximum amount of bioactive compounds and increase the overall extraction efficiency.

### Gas chromatography-mass spectrometry

**Fig. 2:**
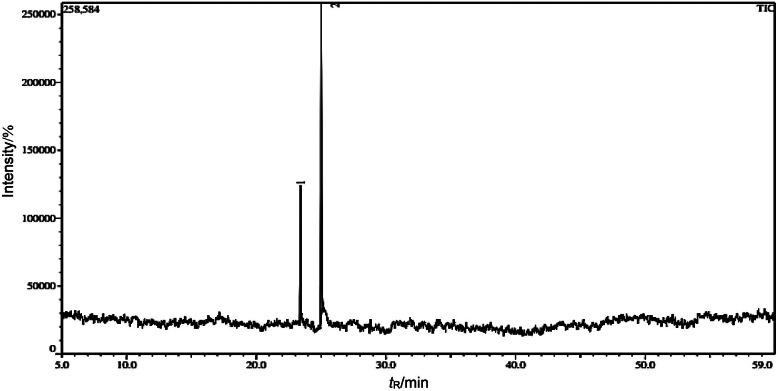
Gas chromatogram of black cumin seed extract showing presence of thymoquinone

The gas chromatography-mass spectrometry (GC-MS) of the methanol extract from black cumin seeds showed the presence of thymoquinone and thymohydroquinone. Thymoquinone was the primary volatile component, accounting for 28.62 %, while thymohydroquinone was tentatively characterised in extracts from black cumin seeds (Fig. 2). In a C18 stationary phase, the gas chromatogram showed two main peaks that were separated after 59 min of run time. The most prevalent compounds were thymohydroquinone (C_10_H_14_0_2_) and thymoquinone (C_10_H_12_0_2_). These compounds were described using accurate *m*/*z=*166.22 and 164.20 mass fragmentation patterns of ion peaks (*31*).

### Fourier transform infrared spectroscopy

**Fig. 3:**
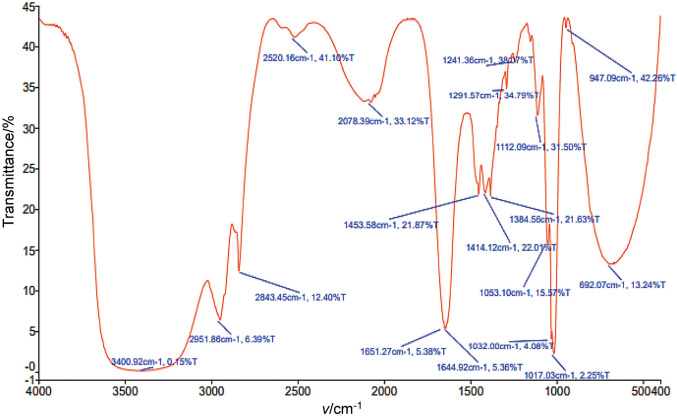
Results of Fourier transform infrared spectroscopy (FTIR) analysis

The Fourier transform infrared spectroscopy (FTIR) spectrum of the methanol solvent extract (Fig. 3) shows a broad absorption band at 3400.92 cm^−1^, indicating the presence of N–H stretching vibrations. The peaks observed between 3000 and 2800 cm^−1^ correspond to the stretching vibrations of isopropyl and -CH_3_ groups in thymoquinone derivatives. Specifically, the peak at 2853.87 cm^−1^ represents the symmetric stretching of the three methyl groups, while the peak at 2923.68 cm^−1^ is associated with C-H stretching in the tertiary carbon of the isopropyl group. Studies by Sopyan *et al.* (*32*) confirm that thymoquinone has distinct FTIR absorption bands at 2924 and 2854 cm^−1^, further supporting these peak designations to thymoquinone derivatives. These findings are consistent with the results of Demirbolat *et al.* (*33*). Additional bands corresponding to C–O stretching vibrations, associated with alcohols, esters and ethers, were observed in the range of 1112.94 to 780.06 cm^−1^.

### Response surface methodology

In ultrasound-assisted extraction, a face-centred central composite design (CCD) was used to optimise the integration of independent variables, namely A: time (30–60 min), B: the ratio of mass of seed powder to volume of solvent (50–100 %) and C: amplitude (30–60 %) - with dependent variables: DPPH activity (%), total phenolic content as *w*(GAE)/(mg/g), and extraction yield (%). The experimental design is shown in Table 1. The results present the optimised and analysed factors (time, the ratio of the mass of seed powder to the volume of solvent, and amplitude) in relation to the response variables (DPPH activity, total phenolic content, and yield). A total of 20 experimental trials, including 6 replications, were conducted using a central composite design to generate various combinations of these factors. Table 2 shows the results of the analysis of variance (ANOVA), which includes an evaluation of the fit of the model, statistical significance and regression coefficients. In addition to the ANOVA results, several statistical parameters were used to assess the accuracy and reliability of the model. The analysis included the coefficient of determination (R^2^), modified R^2^ and coefficient of variation (CV), all of which are crucial for assessing the fit of the model to the experimental data and the variability of the responses. The correlation coefficients (R^2^) for the three were determined to be 0.980, 0.948 and 0.903, respectively. The high R^2^ values indicate that the models describe the data effectively and are reliable for predicting system behaviour. The R^2^ value is crucial for evaluating the quality of fit, with values approaching 1 indicating a better fit between the model and the observed data.

**Table 2:** ANOVA results of ultrasound-assisted extraction of thymoquinone from methanol extracts

Parameter	DPPH scavenging activity/%	TPC as *w*(GAE)/(mg/g)	*Y*/%
	F-value		
Model	37.09***	13.31***	16.78***
A: *t*/min	19.17***	21.15***	19.43***
B: (*m*(seed powder)/*V*(solvent)/)%	14.84***	14.7***	11.41**
C: Amplitude/%	23.0***	12.30**	12.50**
AB	33.0***	49.20***	10.04 **
AC	63.50***	70.58***	11.51**
BC	61.75***	80.42***	21.21***
A^2^	75.01***	11.67**	43.38***
B^2^	79.62***	4.48 ns	63.73***
C^2^	84.67***	8.62*	49.0***
Residual	21.90	17.97	10.21
Lack-of-fit	0.584 ns	0.813 ns	0.565 ns
R^2^	0.980	0.948	0.903
R^2^ (adjusted)	0.954	0.922	0.890

DPPH=2,2-diphenyl-1-picrylhydrazyl radical, TPC=total phenolic content, *Y*=yield. All the linear and quadratic values are significant and lack-of-fit was not significant; ns=not significant (p>0.05), *significant at (p<0.05), **significant at (p<0.01), ***significant at (p<0.0001)

**Fig. 4:**
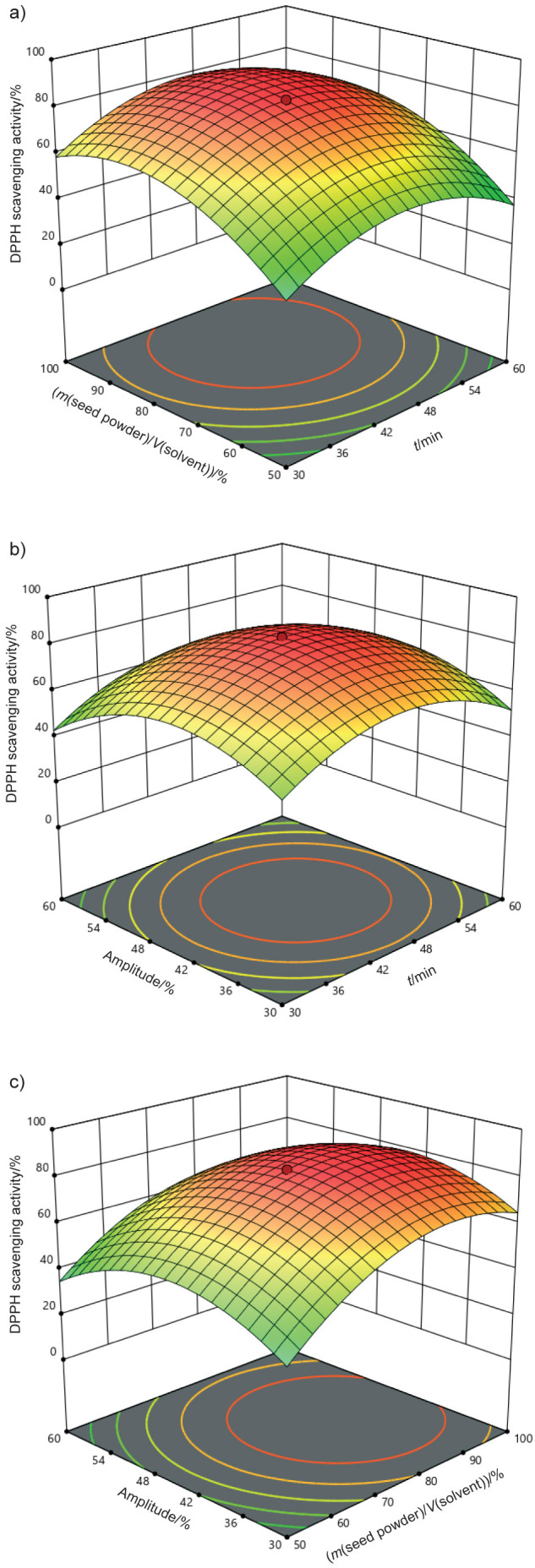
3D surface plots illustrating the synergistic impact of process factors for DPPH scavenging activity: a) ratio of mass of seed powder to volume of solvent *vs.* time, b) amplitude *vs.* time and c) amplitude *vs*. ratio of mass of seed powder to volume of solvent

**Fig. 5:**
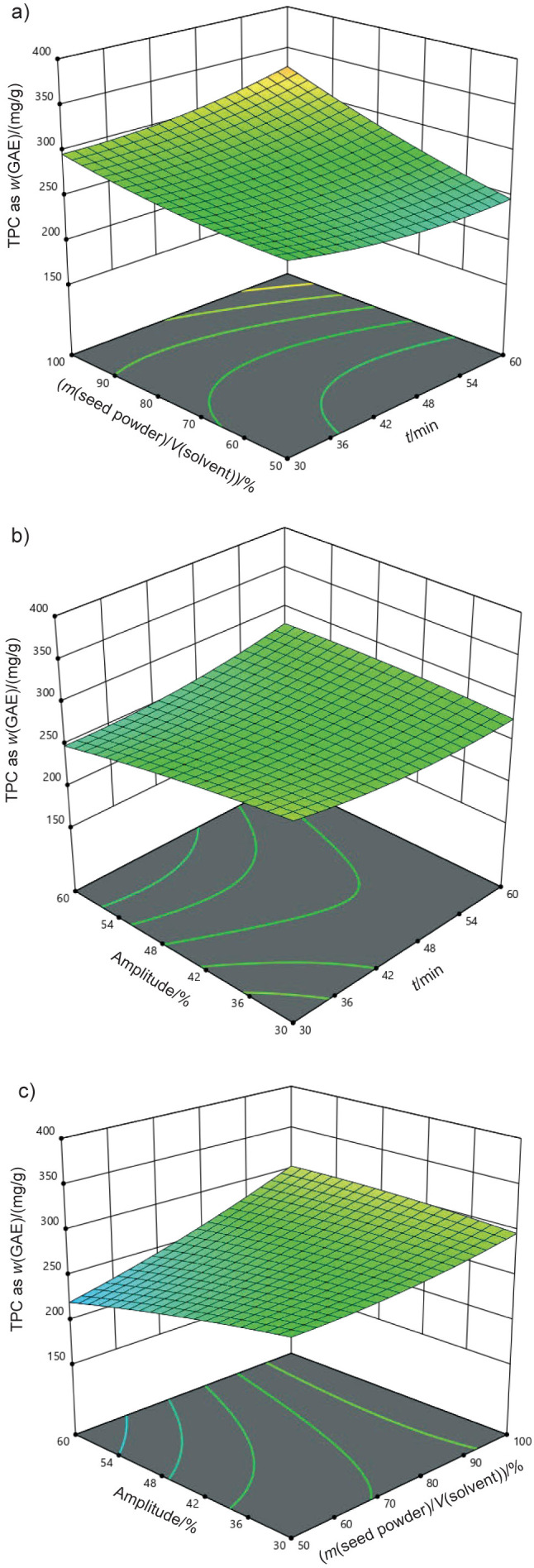
3D surface plots illustrating the synergistic impact of process factors for total phenolic content (TPC) as *w*(GAE)/(mg/g) as a function of: a) ratio of mass of seed powder to volume of solvent *vs.* time, b) amplitude *vs.* time and c) amplitude *vs*. ratio of mass of seed powder to volume of solvent

**Fig. 6:**
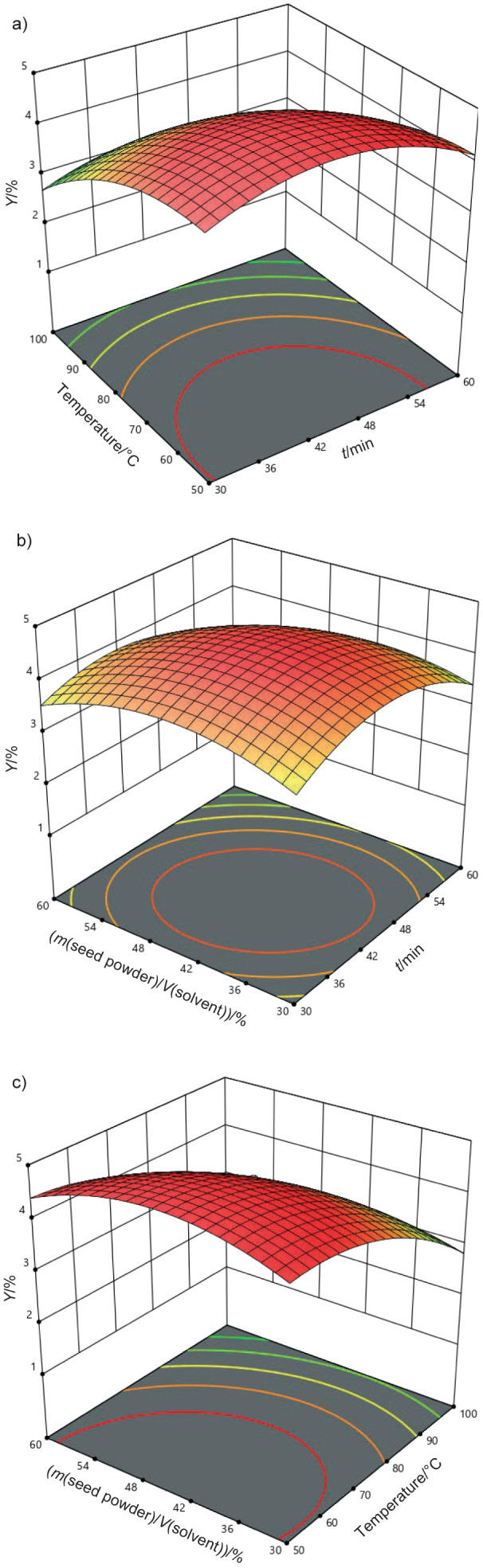
3D surface plots illustrating the synergistic impact of process factors of yield (*Y*) as a function of: temperature *vs*. time, b) ratio of mass of seed powder to volume of solvent *vs.* time and c) ratio of mass of seed powder to volume of solvent *vs*. temperature

The F-values obtained for the models for DPPH, TPC as GAE, and yield were 37.09 %, 13.31 mg/g and 16.78 %, respectively, all of which were statistically significant. The F-value is a critical parameter of ANOVA as it indicates the ratio of variance explained by the model compared to the unexplained variance. Higher F-values indicate a stronger and more reliable model. In this particular instance, the significant F-values for each response demonstrate that the models successfully capture the basic correlations between the extraction parameters and the recovery of the bioactive compounds (*34*, *35*). The reliability of the optimisation of the extraction using the fitted RSM depends on the strength of the fit of the model. This is determined by both the adequacy of the model and the statistical significance of the F-values. The high R^2^ values and significant F-values indicate that the models are robust and can be confidently used for further optimisation of the ultrasound-assisted extraction. These results demonstrate the importance of using appropriate statistical tools such as ANOVA and response surface methodology (RSM) to optimise extraction conditions to maximise the recovery of bioactive compounds from black cumin seeds. The 3D surface plots shown in Fig. 4, Fig. 5 and Fig. 6 were used to evaluate the adequacy of the model and to investigate the relationships between the experimental variables studied. These plots visually represent the correlation between the independent factors and the response variables, and provide valuable insight into how the extraction parameters influence the results. The results clearly showed that the variables involved in the extraction process - such as extraction time, solvent volume, and amplitude - significantly affected the response variables, namely antioxidant activity, total phenolic content (TPC) and yield (*36*). The study showed that interactions between the extraction time and the ratio of mass of seed powder to volume of solvent (A, B), amplitude and time (C, A), and solvent volume and amplitude (B, C), had a significant effect on the extraction results. Specifically, the antioxidant activity was significantly increased under three conditions: a methanol volume of approx. 75 %, an extraction time of 30–45 min and an amplitude setting of 30–45 %. These results suggest that optimising these parameters can significantly improve the yield of bioactive compounds, especially those with antioxidant properties. The results of previous studies, such as by Chakraborty *et al.* (*37*), confirm that the ultrasound-assisted extraction (UAE) promotes the dissolution of phenolic compounds, with higher ethanol amount contributing to more efficient extraction. As the ethanol content increases, the solubility of the phenolic compounds improves, which increases the overall antioxidant activity of the extracts. The interaction of key factors in UAE highlights the importance of optimising these parameters to achieve the best possible results in terms of both yield and antioxidant potential.

When water and ethanol are combined, water acts as a swelling agent, while ethanol plays a key role in breaking the bonds that hold solutes in the cellular matrix. This facilitates the extraction of bioactive compounds (*38*, *39*), especially thymoquinone, which is known for its strong antioxidant activity (*40*). A higher amount of solvent improves the extraction by enhancing the dissolution of these compounds. However, the optimal ratio of mass of seed powder to volume of solvent to extract thymoquinone was found to be 75 %, especially in combination with an extraction time of 45 min. Under these conditions, the total phenolic content reached a moderate level, while the yield was 4.5 %, indicating that these parameters allow the most efficient extraction. Similar results have been reported in other studies (*41*-*43*), confirming that the extraction of bioactive compounds is possible using optimised methods that effectively preserve their antioxidant and therapeutic properties. In the current study, the thymoquinone content of 28.67 % was found in the methanol extract, a result consistent with previous studies (*44*, *45*) that reported a thymoquinone content of 22 %. These results emphasise the efficacy of ultrasound-assisted extraction and the use of methanol as an optimal solvent to maximise thymoquinone yield from black cumin seeds.

## CONCLUSIONS

In the current study, the extraction conditions for bioactive compounds were optimised using the response surface approach. The antioxidant activity, total phenolic content and yield of black cumin extract were analysed. Improved extraction efficiency for thymoquinone (28.62 %) was also achieved using an ultrasound-assisted extraction method. Ultrasonic extraction was optimised using a three-factor and three-response design, where quadratic polynomial statistical models were developed and validated to improve thymoquinone extraction. The results of the study showed that the efficiency of thymoquinone extraction reached 28.62 % when ultrasonic extraction was carried out with methanol as a solvent. Total phenolic content (TPC) as GAE, yield and DPPH radical scavenging activity were improved to 271.03 mg/g, 4.5 % and 83.06 %, respectively. Thymoquinone content was significantly affected by both amplitude and the ratio of the mass of seed powder to volume of solvent. Additionally, the improved extraction efficiency of ultrasonic extraction was confirmed by SEM image analysis, which showed clear disruption and disintegration of the matrix cell wall. GC-MS and FTIR analyses confirmed the predominant presence of thymoquinone and thymohydroquinone. Consequently, black cumin seeds are considered a rich source of phenolic compounds and thymoquinone, which contribute significantly to antioxidant activity. The novelty of this work lies in the detailed statistical optimisation approach and validation of ultrasound-assisted extraction, demonstrating its superiority in disrupting the cell wall matrix, as confirmed by SEM analysis. The application of GC-MS and FTIR analyses also showed the dominance of thymoquinone and thymohydroquinone in the extracts, further emphasising *Nigella sativa* as a rich source of phenolic compounds with strong antioxidant activity. This study contributes to advancing green extraction technologies and promotes the use of *Nigella sativa* as a sustainable source for the development of nutraceuticals and functional foods. The results provide a promising basis for the large-scale production of extracts rich in bioactive compounds and their potential application in the health and pharmaceutical industries. Building on the results of this research, future studies could investigate the encapsulation and formulation of thymoquinone-enriched extracts to improve bioavailability and stability. In addition, investigating the therapeutic efficacy of these extracts in clinical settings in the treatment of oxidative stress, inflammation and immune-related disorders will further validate their potential. Extending this work to include other bioactive compounds from *Nigella sativa* and exploring synergistic effects with other natural antioxidants could lead to novel functional food and pharmaceutical innovations.

## References

[1] Ahmad A Husain A Mujeeb M Khan SA Najmi AK Siddique NA A review on therapeutic potential of *Nigella sativa*: A miracle herb. Asian Pac J Trop Biomed. 2013;3(5):337–52. 10.1016/S2221-1691(13)60075-1 23646296 PMC3642442

[2] Ahmad A Mishra RK Vyawahare A Kumar A Rehman MU Qamar W Thymoquinone (2-Isopropyl-5-methyl-1, 4-benzoquinone) as a chemopreventive/anticancer agent: Chemistry and biological effects. Saudi Pharm J. 2019;27(8):1113–26. 10.1016/j.jsps.2019.09.008 31885471 PMC6921197

[3] Alam I Shahi NC Lohani UC Kumar A Prakash O. Ultrasound assisted extraction of oil from black cumin (*Nigella sativa* L.). Int J Chem Stud. 2021;9(2):87–91. 10.22271/chemi.2021.v9.i2b.11698

[4] Kaushik N Barmanray A. A study on physico-chemical properties and nutritional profile of an indigenous cultivar - Black cumin (*Nigella sativa* L.). Int J Food Nutri Sci. 2022;11 S1:379–91.

[5] Dubey PN Singh B Mishra BK Kant K Solanki RK. Nigella (*Nigella sativa*): A high value seed spice with immense medicinal potential. Indian J Agric Sci. 2016;86(8):967–79. 10.56093/ijas.v86i8.60500

[6] Nyemb JN Shaheen H Wasef L Nyamota R Segueni N Batiha GES. Black cumin: A review of its pharmacological effects and its main active constituent. Pharmacol Rev. 2022;16(32):107–25. 10.5530/phrev.2022.16.16

[7] Kaushik N Barmanray A. Solvent selection for efficient extraction, GC-MS and FT-IR characterization of major bioactive compounds present in different seed extracts of *Nigella sativa* L. Eur Chem Bull. 2023;12(13):836–59.

[8] Kaushik N Barmanray A. Evaluation of flavonoids content, phenolic compounds and antioxidant properties of *Nigella sativa* seed extracts using different solvents. Int J Food Nutri Sci. 2022;11 S3:2964–73.

[9] Kiralan M Özkan G Bayrak A Ramadan MF. Physicochemical properties and stability of black cumin (*Nigella sativa*) seed oil as affected by different extraction methods. Ind Crops Prod. 2014;57:52–8. 10.1016/j.indcrop.2014.03.026

[10] Chemat F. Zill-E-Huma, Khan MK. Applications of ultrasound in food technology: Processing, preservation and extraction. Ultrason Sonochem. 2011;18(4):813–35. 10.1016/j.ultsonch.2010.11.023 21216174

[11] Sharma P, Longvah T. Nigella (*Nigella sativa*) seed. In: Tanwar B, Goyal A, editors. Oilseeds: health attributes and food applications. Singapore, Singapore: Springer; 2021. pp. 331-50. 10.1007/978-981-15-4194-0_13

[12] Hannan MA Rahman MA Sohag AAM Uddin MJ Dash R Sikder MH Black cumin (*Nigella sativa* L.): A comprehensive review on phytochemistry, health benefits, molecular pharmacology, and safety. Nutrients. 2021;13(6):1784. 10.3390/nu13061784 34073784 PMC8225153

[13] Ambati RR, Ramadan MF. *Nigella sativa* seed extracts in functional foods and nutraceutical applications. In: Ramadan MF, editor. Black cumin (*Nigella sativa*) seeds: Chemistry, technology, functionality, and applications. Food bioactive ingredients. Cham, Switzerland: Springer Nature; 2021. pp. 501-20. 10.1007/978-3-030-48798-0_31

[14] Kabir Y Shirakawa H Komai M. Nutritional composition of the indigenous cultivar of black cumin seeds from Bangladesh. Prog Nutr. 2019;21 1-S:428–34. 10.23751/pn.v21i1-S.6556

[15] Salehi B Quispe C Imran M Ul-Haq I Živković J Abu-Reidah IM *Nigella* plants–Traditional uses, bioactive phytoconstituents, preclinical and clinical studies. Front Pharmacol. 2021;12:625386. 10.3389/fphar.2021.625386 33981219 PMC8107825

[16] Benazzouz-Smail L Achat S Brahmi F Bachir-Bey M Arab R Lorenzo JM Biological properties, phenolic profile, and botanical aspect of *Nigella sativa* L. and *Nigella damascena* L. seeds: A comparative study. Molecules. 2023;28(2):571. 10.3390/molecules28020571 36677629 PMC9863492

[17] Perera WPRT Liyanage JA Dissanayake KGC Chandrasiri WAL Gunathilaka H. A review on pharmacological activities and anti-microbial properties of *Nigella sativa* and isolated thymoquinone. J Med Plants Stud. 2021;9(3):118–22. 10.22271/plants.2021.v9.i3b.1277

[18] Niu Y Zhou L Meng L Chen S Ma C Liu Z Recent progress on chemical constituents and pharmacological effects of the genus *Nigella.* Evid Based Complement Alternat Med. 2020;2020:6756835. 10.1155/2020/6756835 32655665 PMC7321528

[19] Rani R Dahiya S Dhingra D Dilbaghi N Kim KH Kumar S. Improvement of antihyperglycemic activity of nano-thymoquinone in rat model of type-2 diabetes. Chem-Biol Interact. 2018;295:119–32. 10.1016/j.cbi.2018.02.006 29421519

[20] Yimer EM Tuem KB Karim A Ur-Rehman N Anwar F. *Nigella sativa* L. (black cumin): A promising natural remedy for wide range of illnesses. Evid Based Complement Alternat Med. 2019;2019:1528635. 10.1155/2019/1528635 31214267 PMC6535880

[21] Solati Z Baharin BS Bagheri H. Antioxidant property, thymoquinone content and chemical characteristics of different extracts from *Nigella sativa* L. seeds. J Am Oil Chem Soc. 2014;91(2):295–300. 10.1007/s11746-013-2362-5

[22] Tiji S Benayad O Berrabah M El Mounsi I Mimouni M. Phytochemical profile and antioxidant activity of *Nigella sativa* L. growing in Morocco. Scie World J. 2021;2021:6623609. 10.1155/2021/6623609 33986636 PMC8079191

[23] Dalli M Azizi SE Kandsi F Gseyra N. Evaluation of the *in vitro* antioxidant activity of different extracts of *Nigella sativa* L. seeds, and the quantification of their bioactive compounds. Mater Today Proc. 2021;45(Part 8):7259–63. 10.1016/j.matpr.2020.12.743

[24] Sanket S Sharma PK Mani I Nain L Satheesh N. Optimization of ohmic parameters in enzyme assisted aqueous extraction for better physico-chemical properties of the black cumin seed oil. Ind Crops Prod. 2024;208:117892. 10.1016/j.indcrop.2023.117892

[25] Khalid A Bashir S Khalil AA Shah FUH Khan AA Arslan Khan M Varietal comparison of proximate analysis and mineral composition of black cumin seed powder. Pak J Food Sci. 2019;29(2):5–8.

[26] Muzolf-Panek M Stuper-Szablewska K. Comprehensive study on the antioxidant capacity and phenolic profiles of black seed and other spices and herbs: Effect of solvent and time of extraction. J Food Meas Charact. 2021;15(5):4561–74. 10.1007/s11694-021-01028-z

[27] Nivetha K Prasanna G. GC-MS and FT-IR analysis of *Nigella sativa* L. seeds. Int J Adv Res Biol Sci. 2016;3(6):45–54.

[28] Mohammed NK Manap MYA Tan CP Muhialdin BJ Alhelli AM Meor Hussin AS. The effects of different extraction methods on antioxidant properties, chemical composition, and thermal behavior of black seed (*Nigella sativa* L.) oil. Evid Based Complement Alternat Med. 2016;2016:6273817. 10.1155/2016/6273817 27642353 PMC5015008

[29] Samaram S Mirhosseini H Tan CP Ghazali HM Bordbar S Serjouie A. Optimization of ultrasound-assisted extraction of oil from papaya seed by response surface methodology: Oil recovery, radical scavenging antioxidant activity, and oxidation stability. Food Chem. 2015;172:7–17. 10.1016/j.foodchem.2014.08.068 25442517

[30] Design-Expert, v. 13.0, StatEase, Minneapolis, MN, USA; 2021.

[31] Šibul FS Orčić DZ Svirčev E Mimica-Dukić NM. Optimization of extraction conditions for secondary biomolecules from various plant species. Hem Ind. 2016;70(4):473–83. 10.2298/HEMIND150531053S

[32] Sopyan I Gozali D. Sriwidodo, Guntina RK. Design-Expert software (DOE): An application tool for optimization in pharmaceutical preparations formulation. Int J Appl Pharm. 2022;14(4):55–63. 10.22159/ijap.2022v14i4.45144

[33] Demirbolat I Kartal M Karik Ü. Development and validation of a GC-FID method to quantify thymoquinone in black cumin seed oils. J Res Pharm. 2019;23(3):506–13. 10.12991/jrp.2019.157

[34] Feng Y Dunshea FR Suleria HAR. LC-ESI-QTOF/MS characterization of bioactive compounds from black spices and their potential antioxidant activities. J Food Sci Technol. 2020;57(12):4671–87. 10.1007/s13197-020-04504-4 33087978 PMC7550543

[35] Prasad KN Hassan FA Yang B Kong KW Ramanan RN Azlan A Response surface optimisation for the extraction of phenolic compounds and antioxidant capacities of underutilised *Mangifera pajang* Kosterm. peels. Food Chem. 2011;128(4):1121–7. 10.1016/j.foodchem.2011.03.105

[36] Almusallam IA Ahmed IAM Babiker EE Al Juhaimi FY Fadimu GJ Osman MA Optimization of ultrasound-assisted extraction of bioactive properties from date palm (*Phoenix dactylifera* L.) spikelets using response surface methodology. LWT – Food Sci Technol. 2021;140:110816. 10.1016/j.lwt.2020.110816

[37] Chakraborty S Uppaluri R Das C. Optimization of ultrasound-assisted extraction (UAE) process for the recovery of bioactive compounds from bitter gourd using response surface methodology (RSM). Food Bioprod Process. 2020;120:114–22. 10.1016/j.fbp.2020.01.003

[38] Azmir J Zaidul ISM Rahman MM Sharif KM Mohamed A Sahena F Techniques for extraction of bioactive compounds from plant materials: A review. J Food Eng. 2013;117(4):426–36. 10.1016/j.jfoodeng.2013.01.014

[39] Moghimi M Farzaneh V Bakhshabadi H. The effect of ultrasound pretreatment on some selected physicochemical properties of black cumin (*Nigella sativa*). Nutrire. 2018;43:18. 10.1186/s41110-018-0077-y

[40] Fadimu GJ Ghafoor K Babiker EE Al-Juhaimi F Abdulraheem RA Adenekan MK. Ultrasound-assisted process for optimal recovery of phenolic compounds from watermelon (*Citrullus lanatus*) seed and peel. J Food Meas Charact. 2020;14(3):1784–93. 10.1007/s11694-020-00426-z

[41] Gueffai A Gonzalez-Serrano DJ Christodoulou MC Orellana-Palacios JC Ortega MLS Ouldmoumna A Phenolics from defatted black cumin seeds (*Nigella sativa* L.): Ultrasound-assisted extraction optimization, comparison, and antioxidant activity. Biomolecules. 2022;12(9):1311. 10.3390/biom12091311 36139150 PMC9496517

[42] Şahin S Şamlı R. Optimization of olive leaf extract obtained by ultrasound-assisted extraction with response surface methodology. Ultrason Sonochem. 2013;20(1):595–602. 10.1016/j.ultsonch.2012.07.029 22964032

[43] Brahmi F Blando F Sellami R Mehdi S De Bellis L Negro C Optimization of the conditions for ultrasound-assisted extraction of phenolic compounds from *Opuntia ficus-indica* [L.] Mill. flowers and comparison with conventional procedures. Ind Crops Prod. 2022;184:114977. 10.1016/j.indcrop.2022.114977

[44] Hameed S Imran A Nisa MU Arshad MS Saeed F Arshad MU Characterization of extracted phenolics from black cumin (*Nigella sativa* Linn), coriander seed (*Coriandrum sativum* L.), and fenugreek seed (*Trigonella foenum-graecum*). Int J Food Prop. 2019;22(1):714–26. 10.1080/10942912.2019.1599390

[45] Yousuf O Gaibimei P Singh A. Ultrasound assisted extraction of oil from soybean. Int J Curr Microbiol Appl Sci. 2018;7(7):843–52. 10.20546/ijcmas.2018.707.103

